# Statistic Complexity: Combining Kolmogorov Complexity with an Ensemble Approach

**DOI:** 10.1371/journal.pone.0012256

**Published:** 2010-08-26

**Authors:** Frank Emmert-Streib

**Affiliations:** Computational Biology and Machine Learning, Center for Cancer Research and Cell Biology, School of Medicine, Dentistry and Biomedical Sciences, Queen's University Belfast, Belfast, United Kingdom; University of East Piedmont, Italy

## Abstract

**Background:**

The evaluation of the complexity of an observed object is an old but outstanding problem. In this paper we are tying on this problem introducing a measure called *statistic complexity*.

**Methodology/Principal Findings:**

This complexity measure is different to all other measures in the following senses. First, it is a bivariate measure that compares two objects, corresponding to pattern generating processes, on the basis of the *normalized compression distance* with each other. Second, it provides the quantification of an error that could have been encountered by comparing samples of finite size from the underlying processes. Hence, the *statistic complexity* provides a statistical quantification of the statement ‘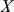 is similarly complex as 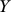
’.

**Conclusions:**

The presented approach, ultimately, transforms the classic problem of assessing the complexity of an object into the realm of statistics. This may open a wider applicability of this complexity measure to diverse application areas.

## Introduction

Complex systems is the study of interactions of simple building blocks that result in a collective behavior or properties absent in the elementary components of the system itself. Due to the fact that this problem does not fit into one of the traditional research fields, it is connected to various of these, for instance physics, biology, chemistry or econometrics [1]–[5]. Many measures, properties or characteristics of a multitude of different complex systems from these fields has been studied to date [6]–[8], however, the *complexity* of an object may have received the most attention. This property of complex systems has fascinated generations of scientists [9]–[11] trying to quantify such a notation. Very coarsely speaking, *an object is said to be ‘complex’ when it does not match patterns regarded as simple*, as López-Ruiz et al. [12] describe it in their article. Over the last decades, many approaches have been suggested to define the complexity of an object quantitatively [9], [11], [13]–[19]. An intrinsic problem with such a measure is that there are various ways to perceive and, hence, characterize complexity leading to complementing complexity measures [20]. For example, Kolmogorov complexity [9], [11], [21] is based on algorithmic information theory considering objects as individual symbol strings, whereas the measures *effective measure complexity* (EMC) [17], *excess entropy* [22], *predictive information* [23] or *thermodynamic depth* [18] relate objects to random variables and are ensemble based. Interestingly, despite considerable differences among all these complexity measures 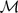 they all have in common that they assign a complexity value to each individual object 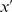 under consideration, 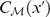. In this paper we will assume that 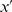 corresponds to a string sequence of a certain length and its components assume values from a certain domain, e.g., 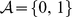 or 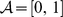. It is of importance to note that there is a conceptually different measure recently introduced by Vitányi et al. that evaluates the complexity *distance* among two objects 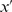 and 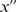 instead of their absolute values. This measure is called the *normalized compression distance* (NCD) [24], 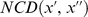, and is based on Kolmogorov complexity [10].

The purpose of this paper is to introduce a new measure of complexity we call *statistic complexity* that is not only different to all other complexity measures introduced so far, but also connects directly to statistics, specifically, to statistical inference [25], [26]. More precisely, we introduce a complexity measure with the following properties. First, the measure is bivariate comparing two objects, corresponding to pattern generating processes, on the basis of the *normalized compression distance* with each other. Second, this measure provides the quantification of an error that could have encountered by comparing samples of finite size from the underlying processes. Hence, the *statistic complexity* provides a statistical quantification of the statement ‘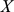 is similarly complex as 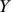
’.

This paper is organized as follows. In the next section we describe the general problem in more detail and introduce our complexity measure. Then we present numerical results and provide a discussion. We finish with conclusions and an outlook.

## Methods

Currently, a commonly acknowledged, rigorous mathematical definition of the complexity of an object is not available. Instead, when complexity measures are suggested they are normally assessed by their behavior with respect to three qualitative patterns, namely simple, random (chaotic) and complex patterns. Qualitatively, a complexity measure is considered *good* if: (1) the complexity of simple and random objects is less than the complexity value of complex objects [17], (2) the complexity of an object does not change if the system size changes. For example, Kolmogorov complexity has the desireable property to remain unchanged if the system size doubles, i.e., 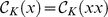, however, it cannot distinguish random from complex pattern because in both cases the compressibility of an object is low resulting in high values of 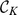. We want to add a third property to the above criteria: (3) A complexity measure should quantify the uncertainty of the complexity value. As motivation for this property we just want to mention that there is a crucial difference between an observed object 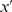 and its generating process 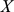 [23]. If the complexity of 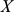 should be assessed, based on the observation 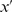 only, this assessment may be erroneous. This error may stem from the limited (finite) size of observations. Also, the possibility of measurement errors would be another source derogating the ability of an error-free assessment. For this reason, the major objective of this article is to introduce a complexity measure possessing all three properties listed above that assesses the complexity classes of the underlying processes instead of individual objects.

We start by pointing out that criteria (1) provides a relative statement connecting different objects. That means the complexity of an object is always related to the complexity of another object [20] leading to relative statements like ‘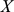 is similarly complex as 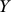
’. Hence, a numerical value 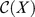 without knowledge of any other complexity value for other objects has no meaning at all. For reasons of mathematical rigor, we propose to include this implicit reference point into a proper definition of complexity. This implies that a fundamental complexity measure needs to be bivariate, 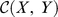, instead of univariate comparing two processes 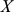 and 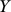. As a side note, we remark that all complexity measures suggested so far we are aware of are univariate measures [13], [14], [16]–[18], [22], [23] with respect to the context set above, except for the normalized compression distance (NCD) [24], [27]. However, a practical problem of the NCD is that Kolmogorov complexity, on which it is based, is not computable but only upper semi-computable [27]. Li et al. introduced in [27] a normalized and universal metric called normalized information distance (NID) which can be approximated by,
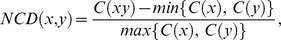
(1)the normalized compression distance [27]. Here, 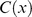 denotes the compression size of string 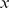 and 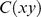 the compression size of the concatenated stings 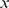 and 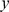. Practically, the quantities 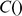 are obtained by compressors like gzip or bzip2, see [28], [29] for details.

**Figure 1:**
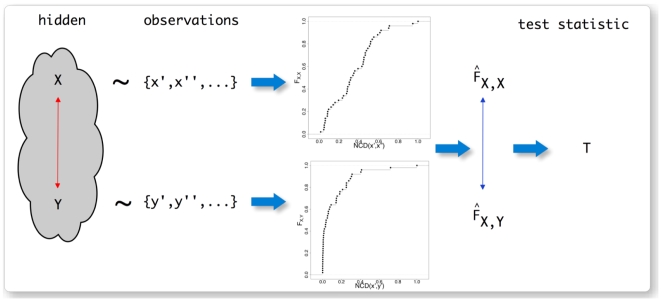
Visualization of the problem and the construction of the test statistic from observations. The double headed arrows represent comparisons of entities. Red indicates that this comparison cannot be performed because the two entities are hidden (unobservable) whereas blue indicates a feasible comparison.

Criteria (3) of a complexity measure stated above acknowledges the fact that an assessment of an object's complexity cannot be without uncertainty or error in case only finite information about this object is available. That means, for a complexity measure to be applicable to real objects (rather than pure mathematical ones) it has to be statistic in order to deal appropriately with incomplete information. Based on these considerations, the *statistic complexity* measure we suggest is defined by the following procedure visualized in Fig. 1:

1. Estimate the empirical distribution function 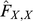 (We indicate estimated entities by 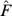 and refer to the ensemble by 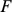.) of the normalized compression distance from 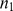 samples, 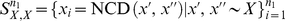, from objects 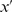 and 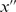 of size 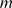 generated by process 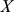 (Here 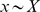 means that 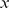 is generated (or drawn) from process (distribution) 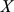.).
2. Estimate the empirical distribution function 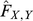 of the normalized compression distance from 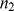 samples, 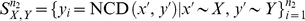, from objects 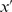 and 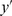 of size 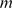 from two different processes, 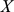 and 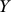.
3. Determine 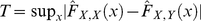 and 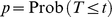.
4. Define, 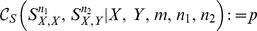, as *statistic complexity*

This procedure corresponds to a two-sided, two-sample Kolmogorov-Smirnov (KS) test [30], [31] based on the normalized compression distance [24], [27] obtaining distances among observed objects. The *statistic complexity* corresponds to the p-value of the underlying null hypotheses, 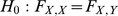, and, hence, assumes values in 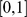. The null hypothesis is a statement about the null distribution of the test statistic 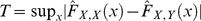, and because the distribution functions are based on the normalized compression distances among objects 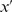 and 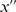, drawn from the processes 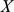 and 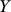, this leads to a statement about the distribution of normalized compression distances. Hence, verbally, 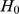 can be phrased as ‘in average, the compression distance of objects from 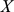 to objects from 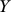 equals the compression distance of objects only taken from 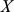
’. It is important to emphasize that this equality holds in *average* and, thus needs to be connected to two ensembles 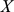 and 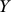. If the alternative hypothesis, 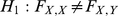, is true this equality does no longer hold implying differences in the underlying processes 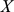 and 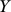, leading to differences in the NCDs. From the formulation of the hypotheses, tested by the *statistic complexity*, it is apparent that we are following closely the guiding principle expressed by López-Ruiz et al. [12] as cited at the beginning of this paper, because 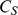 is intrinsically a comparative measure. As a side note regarding the choice of the null hypothesis we want to remark that substituting 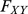 with 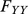 may encounter problems in cases where the complexity value of objects in 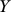 is systematically shifted compared to the complexity value of objects in 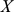. In this case, the distributions 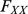 and 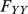 could be similar, although, the complexity of elements in 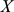 and 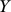 are different. Practically, this may correspond to a pathological case rarely encountered in practice, however, conceptually, such a null hypothesis is apparently less stringent.

Regarding the notation and interpretation of the above procedure it is important to note the following. First, the entities 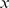 and 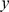 refer to values of the NCD. For example, 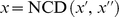 whereas 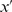 and 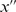 are observable objects that are identically and independently (iid) generated from a process 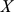, 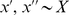. Because 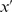 and 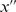 are generated from the same process 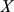, the resulting distribution function 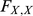 is only indexed by this process. The 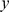 entities are obtained similarly, however, in this case 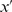 and 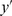 are objects generated from two *different* processes, namely 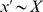 and 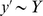. For this reason the distribution function is indexed by these two processes, 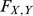. Second, we use the notation, 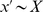, to indicate that 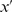 is generated from a process 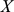, but also that 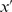 is drawn from 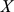. The first meaning is clear if thinking of 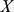 as a model for a complex system, e.g., a cellular automata or a stochastic process. The latter emphasizes the fact that such a process, even if deterministic, becomes random with respect to, e.g., random initial conditions and, hence, effectively is a stochastic process. Third, for reasons of conceptual simplicity we require all objects to have the same size 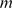. This condition may be relaxed to allow objects of varying sizes but it may require additional technical consideration. On a technical note, the above defined *statistic complexity* has the very desirable property that the power reaches asymptotically 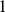 for 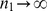 and 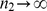 [32]. This means, for infinite many observations the error of the test to falsely accept the null hypotheses when in fact the alternative is true becomes zero. This limiting property is important to hold, because in this case all information about the system is available and, hence, it would be implausible if for such circumstances no error-free decision could be achieved. Formally, this property can be stated as 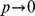 for 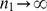 and 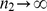. Finally, we would like to note that despite the fact that *statistic complexity* is a statistical test, it borrows part of its strength from the NCD respectively Kolmogorov complexity on which this is based on. Hence, it unites various properties from very different concepts.

## Results

In the following we provide different numerical examples for data frequently used when studying complexity measures. This allows a direct comparison of ours with different measures.

The first characteristic of the *statistic complexity* we study is the influence of the size 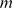 of objects on 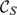. Table 1 shows the results for comparing patterns generated by different rules of one-dimensional cellular automata. Column one represents the reference process, 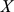, and column two corresponds to 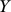. The third and fifty column shows the averaged p-values obtained for cellular automata of length 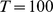 respectively 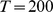 - column four and six provide the variances for the corresponding p-values. For the simulation results shown in Table 1 we generated spatiotemporal patterns for one-dimensional CA for 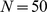 (space) and 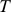 (time), an alphabet of size 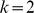 and a 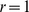 neighborhood with periodic boundary conditions. As burn-in time we used 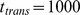 time steps. Each of these spatiotemporal pattern 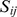, with 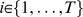 and 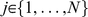, is transformed to its difference pattern 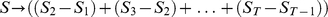 (Here 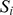 with 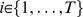 corresponds to a row vector of length 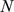.) resulting in a string (object) of length 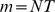 to be applicable for the NCD. Here, the operator 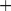 means concatenation of strings. See [29] for numerical details for the application of NCD. The results in Table 1 show that the p-values remain in the same order of magnitude if the size of an object 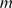 is doubled meaning that the overall quantitative assessment of two processes 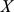 and 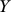 - based on sampled objects thereof - by the measure 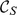 is invariant to extensions of the size 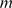. Next we demonstrate that the *statistic complexity* is capable to differentiate between random and complex objects. For this reason we compare rule 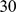, producing random patterns, with rule 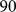, 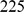, both random, and rule 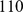, which is complex because it is capable of universal computation. From Table 1 one can see that the p-values correspond with our expectations giving high values for 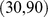 and 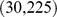 and low values for 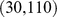. In addition we compare rule 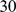 with rules 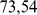 and 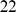, classified according to Wolfram as random, and obtain very low p-values, suggesting significant differences among those patterns. The crucial point here is that not all CA rules that produce chaotic patterns are indistinguishable from each other. In [33] the growth exponent of the roughness along other measures have been used to obtain several subclasses for CA rules leading to chaotic behavior. Comparing our results with their classification reveals that actually rule 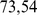 and 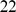 are in different subclasses whereas rule 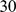 is classified together with rule 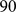 and 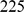. Last, we compare rule 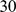 with a periodic pattern, rule 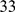, and obtain also in this case a clear distinction. In summary, 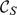 can not only distinguish between simple and complex patterns but finds also meaningful substructures among chaotic patterns if rule 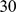 is used as reference process.

**Table 1:** Results for one-dimensional CA (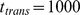, 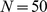, 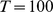 (third and fourth column) and 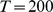 (fifth and sixth column)) averaged over 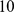 runs.

X	Y	T = 100		T = 200	
CA rule	CA rule	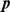	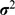	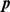	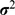
30	30	0.593	0.075	0.684	0.102
30	90	0.617	0.102	0.575	0.139
30	225	0.388	0.131	0.632	0.086
30	73	0.002	0.001	0.002	0.001
30	54	0.002	0.000	0.001	0.001
30	22	0.002	0.000	0.001	0.001
30	33	0.001	0.001	0.002	0.000
30	110	0.002	0.001	0.002	0.001

First column: process 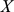. Second column: process 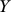. Sample size is 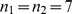.

**Figure 2:**
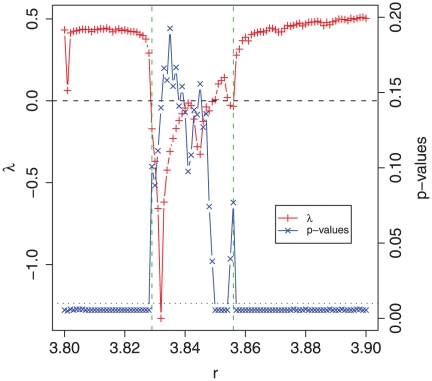
Lyapunov exponent (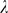 - red line, plus symbol) and p-values (blue line, cross symbol) of the logistic map in dependence on 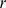. The dotted horizontal line corresponds to a significance level of 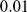 and the dashed line to 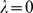.

Next, we apply our measure to the logistic map and compare the results with the Lyapunov exponent (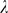). The results are summarized in Fig. 2. We calculate the time series for various values of 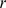 (x-axis) in the intervall 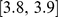 (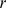 was varied in step sizes of 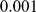 and sample size was 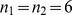.). 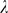 assumes negative values in 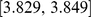 and 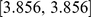 indicating a nonchaotic behavior of the logistic map for these values of 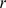. The vertical dashed line separates positive from negative values. The p-values of the *statistic complexity* (blue line, cross symbols) are obtained for each value of 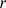 by averaging over 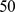 time series each of length 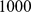 (After waiting a transient period of 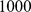 steps.). As reference process, 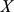, we use a logistic map with 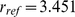, which corresponds to a periodic behavior. From Fig. 2 one can see that there are essentially two types of p-values, ones that are not zero and ones that are close to zero. For example, using a significance level of 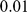 (dotted horizontal line) one obtains that significant values correspond to positive Lyapunov exponents and non-significant values to negative Lyapunov exponents. Again, we want to emphasize that the p-values do not provide a yes or no answer if the logistic map, for a given 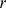 value, is chaotic or nonchaotic but the correct interpretation is that low p-values provide strong evidence against the null hypotheses whereas high p-values do not allow to reject the null hypotheses. Because we use 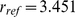 as reference - for which the logistic map shows periodic (nonchaotic) behavior - this is a similar though not identical question. The results for the logistic map allow a comparison with a well studied system. As demonstrated by our results shown in Fig. 2, for an appropriately chosen reference process, 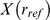, there is a clear correspondence between the *statistic complexity* and the Lyapunov exponent. This property is certainly desirable to hold because it may allows to connect to traditional contributions in the field beyond the logistic map. The possibility of such a connection, despite the seemingly different methods underlying the *statistic complexity* respectively the Lyapunov exponent, can be attributed to the parametric form of our complexity measure allowing a flexibility that is entirely missing in other measures. More importantly, this flexibility is not imposed into the measure but follows naturally from a consequent interpretation of complexity as a referential measure [12] implying imperatively the existence of a reference process 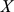 against which another process 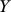 is *quantitatively* compared.

## Discussion

The complexity measure introduced in this paper has several properties that are different to all other measures proposed so far. First, 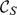 is a bivariate measure allowing to make comparative statements, instead of absolute ones. This may appear as a disadvantage first, however, as López-Ruiz et al. [12] point out, we inevitably compare patterns with each other to make a decision about their complexity (See also the *comparative* discussion on page 909 in [17] about the three patterns shown in Fig. 1.) [20]. Second, we do not make assumptions with respect to the size of patterns to which our measure can be applied, instead, principally, we allow patterns of any finite or infinite size 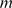. For example, measures like EMC or *excess entropy* are based on block entropies of varying order 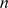 and the final measure is obtained in the limit for 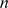 against infinity. Strictly, such measures require an infinite amount of data. Third, due to the fact that *statistic complexity* allows the comparison of patterns of any size 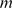 with finite sample sizes 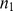 and 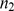 the result of the comparison may be erroneous. The KS test, underlying 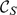, allows a quantification of such an error statistically. Because this error can be quantified in dependence on 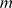, 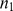 and 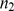, there is no need to assume limiting properties. At this point we would like to re-emphasize that the term *statistic complexity* has been chosen to underline the involvement of a *test statistic* in our measure on which the complexity value is based. For this reason other complexity measures that have been named *statistical complexity* [12], [34], [35] are not similar to our measure at all due to the fact that none of these measures uses a test statistic or a statistical test. Hence, they are actually not related to statistics (the field). An alternative name for these measures would be *probabilistic complexity*, which would make this difference more obvious. The fourth point relates to the empirical distribution functions. The reason for their introduction is, besides the fact that they allow a connection to the KS test, they allow the introduction of two ensembles, one for the process 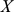 and one for processes 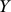. These ensembles compensate that the classic Kolmogorov complexity is not related to any ensemble but only to one string. Further, the ensembles induce a probabilistic interpretation of the deterministic NCD with respect to the underlying processes that generate the patterns. This is in accordance with [17] emphasizing the importance of complexity measures being probabilistic. Taken together, this allows a quantifiable approximation, in dependence on 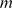, 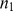 and 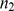, of the underlying processes 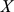 and 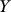 with respect to the information they provide about their complexity, in form of the real observable patterns.

From an applied point of view, the direct connection of *statistic complexity* with statistical inference allows a confirmatory analysis of the complexity of objects. Due to the fact that the uncertainty of a complexity comparison is inherently provided by our measure, it is applicable to (real) objects from a multitude of different application domains. In the future we are planing to investigate the complexity of biological pathways in the context of cancer and other complex diseases [37]. A further potential direction would be an analysis of different *goodness-of-fit* tests. For example, it would be interesting to study a Cramér-von Mises or an Anderson-Darling test, instead of a Kolmogorov-Smirnov test [36]. Other tests may have advantages in different application areas or specific experimental conditions, although, a Kolmogorov-Smirnov test was sufficient with respect to the applications studied in this paper.
